# High-density microarray expression profiling in conventional papillary thyroid carcinomas with versus without a BRAF mutation

**DOI:** 10.1186/1471-2164-15-S2-P21

**Published:** 2014-04-02

**Authors:** Reem Alotibi, Alaa Al-Ahmadi, Manar Ata, Sajjad Karim, Etimad Huwait, Mamdooh Gari, Mohammad Hussain Al-Qahtani, Hans-Juergen Schulten, Jaudah Al-Maghrabi

**Affiliations:** 1 Center of Excellence in Genomic Medicine Research, King Abdulaziz University, Jeddah, Kingdom of Saudi Arabia; 2 Department of Biochemistry, King Abdulaziz University, Jeddah, Kingdom of Saudi Arabia; 3 Department of Pathology, Faculty of Medicine, King Abdulaziz University, Jeddah, Kingdom of Saudi Arabia; 4 Department of Pathology, King Faisal Specialist Hospital and Research Center, Jeddah, Kingdom of Saudi Arabia

## Background

Whereas 40 % to 70 % of papillary thyroid carcinomas (PTCs) are characterized by a BRAF mutation (BRAF^mut^), unified biomarkers for the genetically heterogeneous group of BRAF wild type (BRAF^wt^) PTCs are not established yet [1,2]. Using state-of-the-art technology we compared RNA expression profiles between conventional BRAF^wt^ and BRAF^mut^ PTCs.

## Materials and methods

Affymetrix HuGene 1.0 ST microarrays were used to generate whole transcript expression profiles in 11 BRAF^wt^ and 14 BRAF^mut^ PTCs. A p-value with a false discovery rate (FDR) ≤ 0.05 and a fold change > 2 were used as a threshold of significance for differential expression. Spearman’s correlation as a similarity matrix was utilized for unsupervised two dimensional hierarchical clustering. The BRAF mutational status was surveyed by direct sequencing the hotspot region of BRAF exon 15.

## Results

Hundred eighty transcripts from annotated genes were significantly differentially expressed between BRAF^wt^ and BRAF^mut^ PTCs and unsupervised cluster analysis was able to separate both groups. The most significantly upregulated genes in BRAF^mut^ compared to BRAF^wt^ PTCs include transmembrane 7 superfamily member 4 (TM7SF4, located on 8q23), glutaminase 2 (GLS2; 12q13), ladinin 1 (LAD1; 1q25.1-q32.3), TBC1 domain family, member 2 (TBC1D2; 9q22.33), chromosome 19 open reading frame 33 (C19orf33; 19q13.2), keratin 19 (KRT19; 17q21.2), and poliovirus receptor-related 4 (PVRL4; 1q22-q23.2). The most downregulated genes in BRAF^mut^ PTCs include inositol 1,4,5-triphosphate receptor, type 1 (ITPR1; 3p26-p25), leucine rich repeat and Ig domain containing 2 (LINGO2; 9p21.2-p21.1), solute carrier family 26, member 4 (SLC26A4: 7q31), deiodinase, iodothyronine, type I (DIO1; 1p33-p32), and hepatic leukemia factor (HLF; 17q22). Among differentially expressed microRNAs, mir492 was highly upregulated in BRAF^mut^ PTCs.

## Conclusions

This study provides a detailed overview of differentially expressed genes between BRAF^wt^ vs. BRAF^mut^ conventional PTCs using whole transcript, high-density microarrays. Valuable candidate genes shall be assessed further to identify molecular pathways and molecular biomarkers which distinguish BRAF^wt^ from BRAF^mut^ PTCs.

*This study was supported by King Abdulaziz City for Science and Technology* (*KACST*) *grants 09-BIO707-03 and 09-BIO820-03.*

## References

[B1] Lee JH Lee ES Kim YS Clinicopathologic significance of BRAF V600E mutation in papillary carcinomas of the thyroid: a meta-analysis Cancer 2007 15 38 46 17520704 10.1002/cncr.22754

[B2] Rusinek D Szpak-Ulczok S Jarzab B Gene expression profile of human thyroid cancer in relation to its mutational status Journal of molecular endocrinology 2011 15 R91 103 21798995 10.1530/JME-11-0023

